# Diversification and use of bioenergy to maintain future grasslands

**DOI:** 10.1002/fes3.75

**Published:** 2016-02-16

**Authors:** Iain S. Donnison, Mariecia D. Fraser

**Affiliations:** ^1^ Institute of Biological, Environmental & Rural SciencesAberystwyth UniversityGogerddan CampusAberystwythSY23 3EEUK

**Keywords:** Bioenergy, biorefining, conservation, grasslands, Miscanthus, pasture

## Abstract

Grassland agriculture is experiencing a number of threats including declining profitability and loss of area to other land uses including expansion of the built environment as well as from cropland and forestry. The use of grassland as a natural resource either in terms of existing vegetation and land cover or planting of new species for bioenergy and other nonfood applications presents an opportunity, and potential solution, to maintain the broader ecosystem services that perennial grasslands provide as well as to improve the options for grassland farmers and their communities. This paper brings together different grass or grassland‐based studies and considers them as part of a continuum of strategies that, when also combined with improvements in grassland production systems, will improve the overall efficiency of grasslands as an important natural resource and enable a greater area to be managed, replanted or conserved. These diversification options relate to those most likely to be available to farmers and land owners in the marginally economic or uneconomic grasslands of middle to northern Europe and specifically in the UK. Grasslands represent the predominant global land use and so these strategies are likely to be relevant to other areas although the grass species used may vary. The options covered include the use of biomass derived from the management of grasses in the urban and semi urban environment, semi‐natural grassland systems as part of ecosystem management, pasture in addition to livestock production, and the planting and cropping of dedicated energy grasses. The adoption of such approaches would not only increase income from economically marginal grasslands, but would also mitigate greenhouse gas emissions from livestock production and help fund conservation of these valuable grassland ecosystems and landscapes, which is increasingly becoming a challenge.

## Grasslands

Humankind has been changing the planet and especially its vegetation in a significant way for millennia. This includes the domestication of crops and animals, which enabled early civilization to evolve. Many of these crops and animal species, including, for example, cereals, forage grasses, pigs, and cattle, were native to grasslands and make up modern intensive agriculture. More extensive grasslands, made up of pasture and rangeland, underwent more indirect selection pressure and still represent the dominant global land use type. Since the industrial revolution, humankind has increasingly moved from living in rural‐based societies and economies to urban‐based ones. Our relationship to the countryside has changed and in many parts of the world concerns have moved from productivity to environmental stewardship. This has presented grassland farmers and their communities with significant challenges as incomes fall and rural infrastructure declines. Grassland agriculture is also experiencing the biggest threat to date in terms of loss of land area to other uses, including expansion of the built environment as well as from cropland, forestry, and energy (solar, wind, biofuels). The use of grasslands as a natural resource either in terms of existing vegetation and land cover or planting of new species for bioenergy present an opportunity, and potential solution, to maintaining the broader ecosystem services that perennial grasslands provide as well as improving the options for grassland farmers and their communities. A number of older studies exist on the processing and fractionation of biomass for feed, food, energy, and diversification of grassland products. However, in this paper, the focus is on the more recent literature, and in particular over the last decade, on the use of grasses and grasslands for energy and other nonfood applications by the authors and others. The paper brings together different grass or grassland‐based studies and considers them as part of a continuum of strategies that when also combined with improvements in grassland production systems (Gerssen‐Gondelach et al. 2015) will improve the overall efficiency of grasslands as an important natural resource and enable a greater area to be managed, replanted, or conserved. All these grassland systems are capable of delivering conservation, grazing and/or energy but each system has a different set of features and properties which make them more or less suited to certain locations, farming systems, and end uses (Table 1). The combination of several of these strategies enables farmers and regions to provide energy feedstocks from more marginal land, and even has the potential through remediation to increase food production. This paper therefore explores a number of approaches which seek to maximize individually or collectively the use of grassland as a natural resource in addition to its traditional uses and other ecosystem services. In other words, bioenergy has the potential to be a land management tool for grasslands, helping to protect habitats, livelihoods, as well as contributing to national and global renewable energy and greenhouse gas emission targets.

**Table 1:** Categorization of grassland into types based on species content, location or function; the grassland major and minor outputs; and the challenges; and potential solutions for each type

Grassland type	Major outputs	Minor outputs	Challenges	Solutions
Natural/semi‐natural	Biodiversity and other ecosystem services, landscape	Energy, livestock forage	Abandonment, cost of environmental management	Develop diversification technology options
Urban	Landscape, recreation	Energy, ecosystem function	Cost of establishment and management, pollution and loss through building	Develop diversification technology options
Extensive pasture	Livestock forage, biodiversity and other ecosystem services	Landscape, energy	Environmental impact of ruminant production	New grass varieties and legume varieties, selected breeding of livestock
Intensive pasture	Livestock forage	Landscape, ecosystem function, energy	Environmental impacts of agronomic inputs and ruminant production	New grass and legume varieties, dietary supplements
Energy	Energy and other nonfood uses	Ecosystem function	Cost of establishment and lack of knowledge of the crop	New establishment methods (seed rather than vegetative propagation), agronomy support

## Use of Natural and Semi‐natural Grasslands

Natural and semi‐natural grasslands systems, sometimes referred to as rough grazings or rangeland are important habitats providing a resource for extensive livestock production and a wide number of ecosystem services, including carbon sequestration, biodiversity, water management, landscape, recreation, and leisure. However, reductions in livestock numbers across Europe in response to changes in support mechanisms and other economic pressures have led to abandonment of large areas of poorer quality native plant communities as farmers focus their activities on improved pastures and crops. Left unmanaged, this vegetation becomes characterized by a build‐up of mature and senescent plant material with a high fiber content and relatively low nutrient value. Many of these communities are dominated by plant species rejected by stock (e.g., *Juncus* spp., *Molinia caerulea*,* Deschampsia cespitosa*,* Juniperus* spp., *Genista* spp.) or have become invaded by undesirable or alien species (e.g., *Pteridium*,* Ulex* spp., *Calamagrostis epigejos*,* Solidago* spp., *Lupinus polyphyllus*). However, many of these grasslands by their nature are important habitats and often form part of the Natura 2000 network of protected areas consisting of sites designated by EU Members under the Habitats and Birds Directives (Melts et al. 2014). Increasingly these areas are managed not by grazing but by cutting excess biomass late in the season to maintain species diversity and avoid dominance by a small number of species. In the US bioenergy production from feedstocks grown on marginal or underutilized land, such as those enrolled in the Conservation Reserve Program, has also been shown to provide greenhouse gas benefits and supplement government subsidies (Gelfand et al. 2011; Jungers et al. 2013). This movement is also increasing more globally, with trends in urbanization and rationalization, away from the more isolated rural lifestyles and living. Habitat management is also often important to protect birds or animals, which may require areas of grassland with a short sward height as feeding sites. Conservation organizations which own or manage land are therefore often active in making interventions such as cutting in these habitats that are no longer managed by agriculture. A further challenge is that even when cut as hay or ensiled, this material is of such low nutritional value to animals that it remains unused and is dumped. Therefore, there is an opportunity for bioenergy production from a genuine waste product which if it is not used presents a management and potential environmental risk. In turn it helps avoid the controversy that there is not enough land to grow energy with an increasing world population, and expectations of that population (Ghose 2014).

The value of low‐input high‐diversity mixtures of native grassland perennials for biofuels has also been championed on the basis of greater greenhouse gas reductions and less agrichemical pollution per hectare than can be achieved from corn grain ethanol or soybean biodiesel (Tilman et al. 2006). The material, however, is heterogeneous and very variable from site to site based on the individual species composition in addition to variability caused by local climate and soil types. Bioenergy strategies for the utilization of this resource therefore tend to focus on preprocessing and potentially fractionation. For example, one solution is the integrated generation of solid fuel and biogas from biomass (IBFT; Richter et al. 2010; Bühle et al. 2012) where a hot water pretreatment is used to wash the sugars and corrosive alkaline metals and chlorine concentrations from the biomass. The biomass is then squeezed through a screw press to generate a soluble fraction which can be fermented through anaerobic digestion to make biogas to power the hot water generation, and a press cake which can be further processed by pelletizing for combustion or pyrolysis to bio‐oil and biochar. Fuel quality is significantly influenced by the botanical composition, but the quality can be improved and to an extent controlled by processing (Hengsen et al. 2012). An initial question on such approaches is whether they are predominantly an environmental problem that needs a solution or whether they can address renewable energy targets and carbon dioxide emission targets. A study by Corton et al. (2013) predicted that conservation management in Wales could potentially generate one million tonnes of biomass annually. This equates to the equivalent of 38% of the Welsh transport sector's greenhouse gas (GHG) reduction target for 2020 or a reduction in GHG emissions by 11% of the domestic sector's reduction target for 2020, depending on conversion routes used. Not only do new applications for high diversity low input grasslands provide more options for those managing these established grasslands but also provides incentives to restore such grassland systems including on contaminated land (Zhou et al. 2009).

Historically, moderate levels of animal performance were achieved in many marginal grassland areas, but structural and compositional changes in vegetation following reductions in stocking rates have significantly lowered the production potential and related economic viability of many types of semi‐natural grassland (Dumont et al. 2013). Furthermore, recent research has confirmed that enteric methane emission intensities are greater when animals consume poorer quality indigenous grassland (Fraser et al. 2014), and thus the carbon footprint per kilogram of output is considerably higher compared to more intensive production systems (Edwards‐Jones et al. 2009; Gill et al. 2009). However, there are benefits both for human health and food security from grass‐based meat production, particularly when forages from areas unsuitable for cultivation are turned into human‐edible products (Lind et al. 2009). Mechanical removal of biomass avoided by grazers for bioenergy production should stimulate new plant growth with a higher nutritional value to livestock, in turn improving protein and energy supplies and related production efficiencies. In situations where competitive plant species that are avoided by grazing livestock have become dominant it should also facilitate changes in species balance which reduce foraging time and improve intake potential by increasing the availability of preferred food items. Thus, production of bioenergy and biorenewable products from underutilized native vegetation should complement rather than compete with traditional pastoral uses when undertaken as part of integrated multi‐functional management systems. By stimulating utilization of abandoned or under‐grazed grasslands, this approach would increase the land cover available for pastoral livestock systems and free up scarcer high‐grade land for crop production. Moving livestock production back into areas which have seen agricultural abandonment would enhance food security and development while preserving rural communities and cultural heritage, including landscapes valuable for tourism and recreational activities. Management of areas of flammable dried grass and shrubby vegetation will also reduce fire risk, a constant hazard within certain regions, which carries environmental costs, for example, in terms of soil erosion and CO_2_ emissions, and which has also been exacerbated by reductions in grazing livestock.

## Use of Improved Grasslands, Forage, and Turf

The urban environment including parks, sports pitches, and transport verges are often planted with grasses and these are also cut. As above, this is regarded as a waste product, not so much because it is too indigestible to feed to livestock but because of the risk of contamination. Bioenergy is therefore an ideal technology to make use of this digestible feedstock. Likewise for livestock production, especially in grass‐dominated feeding systems, there can be periods of the year when excess grass can be cut in addition to that needed so that farmers can produce both meat or milk, and energy. This can provide an incentive to optimize grassland system productivity and also help mitigate greenhouse gas emissions from other parts of the farming enterprise. For both urban green waste (Van Meerbeek et al. 2015) and surplus forage, fermentation‐based conversion routes such as anaerobic digestion (Kyazze et al. 2008) and ethanol production (Martel et al. 2010; Farrar et al. 2012) are more likely to be suited to this largely green wet biomass. These feedstocks are also ideally suited for fermentation as a feedstock to a wider range of chemical building blocks or other industrial biotechnology targets (Hull et al. 2014). The high sugar perennial ryegrasses (*Lolium perenne*) have proved to be of particular interest, given the greater availability of readily fermented soluble sugars and principally fructan (Kyazze et al. 2008; Farrar et al. 2012). Once again exploitation of these grasses for bioenergy production could take place as part of management systems integrating this with livestock production. Recent research found that the voluntary intake of lambs was higher when offered silage prepared from grass fiber than when offered silage prepared from unprocessed grass. This was likely due to the physical damage to the grass during processing promoting a more rapid breakdown of the feed in the rumen, leading to a shorter retention time. Related performance figures suggest that these higher voluntary intakes could offset the relatively lower nutritional value of pressed grass, highlighting the potential for simultaneous production of bioenergy and meat/milk from grass, and offering another opportunity to reduce greenhouse gas burdens associated with ruminant production.

Being perennial, forage and turf grasses offer wider ecosystem benefits including carbon sequestration, water management, and soil structure. However, these grasses are often replanted on a medium term (5–10 year) basis and this provides opportunities for replanting with new varieties of forage grasses, such as high sugar types, or the planting of different species of grasses. Potentially these grasses could be nonnative which may provide new opportunities in relation to increases in productivity and also tolerance to changes in climate.

## Use of Dedicated Energy Grasses

Studies on dedicated energy crops have highlighted and calculated: areas of underutilized land (Hastings et al. 2009); the opportunities to be created through plant breeding and agronomy to increase yields and reduce land take (Karp and Shield 2008); fossil carbon substitution and reduction in atmospheric CO_2_ (Hughes et al. 2010); and the provision of wider ecosystem services (Hedde et al. 2013; Bourke et al. 2014). To date, much of the dedicated energy grass science and plant breeding has been focused on grasses such as Miscanthus, switchgrass, napier grass, arundo, and reed canary grass. The advantage of these grasses is that they grow on marginal land and produce high outputs from low inputs. They are typically harvested after senescence and so nitrogen has been remobilized to below ground rhizome and, or, rooting systems. These grasses are also often capable of growing in multiple environments and geographies thereby representing good targets for, and justification of, plant breeding and agronomy research as the markets for seeds or propagules are expanded. The choice of energy grass is largely based on the target environmental conditions (Clifton‐Brown et al. 2011; Don et al. 2012). This applies both to temperatures required for germination, but also for crop growth, over wintering, and then ripening. Miscanthus in particular has received a lot of interest as an energy crop because it combines the high productivity and water and nutrient use efficiencies of a C4 tropical grass with remarkable cold adaptation, with its natural geographic range extending from the tropics in South East Asia up to northern China, Japan, and Siberia. Miscanthus was originally collected by botanists and horticulturalists and brought to Europe and North America from the 19th century onwards (Dougherty et al. 2014). These collections included the naturally occurring triploid hybrid between *M. sinensis* (2×) and *M. sacchariflorus* (4×), *M. x giganteus* (3×). The two parental species and the hybrid have been the subject of studies to understand this species and optimize it for bioenergy production. Research tends to focus on three main areas: (1) yield and yield maintenance; 2) biomass quality; and (3) sustainability and environmental impacts. The key advantage in the development of dedicated energy grasses is that plant breeding and agronomy can be targeted on their specific use for bioenergy, although with quality traits a wider number of markets are developing including for biomaterials, chemicals and even the use for winter animal bedding as a lower cost and local alternative to cereal straws. One major trait of interest has been flowering time because it impacts yield (Jensen et al. 2013) with delayed flowering extending vegetative growth into later in the season (Jensen et al. 2011a); quality through association with the start of senescence; and sustainability and the environment through remobilization of nitrogen following senescence (Mos et al. 2013) and the control of seed dispersal to minimize the risk of invasiveness (Dougherty et al. 2014). In addition, the ability to control flowering enables the crossing of different species that do not necessarily flower at the same time with *M. sacchariflorus* being a short day plant (Jensen et al. 2013) and *M. sinensis* flowering more according to accumulated temperature (Jensen et al. 2011a) with rainfall also having an effect (Jensen et al. 2011b). The generation of seed‐based Miscanthus varieties also has the potential to significantly reduce financial and carbon establishment costs compared to rhizomes.

Strategies to increase yield, beyond the manipulation of flowering time, include extending crop canopy duration, improving net photosynthetic efficiency, incorporating disease, chilling and drought resistance, and improving nutrient and water use efficiencies. Extending the crop canopy duration through earlier emergence in spring and delayed senescence in the autumn, and an efficient crop canopy architecture, represent means to improve the proportion of radiant light intercepted (Robson et al. 2013a). However, extensions of crop canopy duration result in a greater risk of the plant experiencing lethal temperatures. In the case of Miscanthus, the impact of early season frosts can be responded to by the emergence of new shoots assuming sufficient resources have been build up in the previous season and so the negative impact is much lower than in many crop species where the entire crop would be lost because the crop is annual or because the harvested portion is a result of flowering and flower initials are laid down the previous year. Significant differences exist between Miscanthus species and genotypes for drought and freezing tolerance, thereby providing routes to improvement (Clifton‐Brown et al. 2002; Purdy et al. 2013). A number of other single or compound traits have also been evaluated and canopy or plant height is one of the more highly correlated traits to yield (Robson et al. 2013b), whilst nonstructural carbohydrate profiles and ratios between soluble sugars and starch have also being shown to be predictive indicators of future productivity (Purdy et al. 2015).

Strategies to improve energy crop quality depend on the ultimate end use(s). For example at present Miscanthus is predominantly used commercially as a feedstock for heat and power either in large power stations or in smaller community or municipal heating systems. For the thermochemical conversion market, the ideotype is a crop which has low N and P to reduce future fertilizer inputs and low NOx emissions, low K, and Cl to reduce corrosion in boilers, low moisture to minimize drying and reduce spoilage during storage, low ash to reduce slagging and consequent operational downtime, and high processability and calorific value to increase energy density. There is additional interest in the use of Miscanthus as a feedstock for fast pyrolysis (Hodgson et al. 2011) or as a source of sugars for fermentation to transport fuels (Brosse et al. 2012) and through anaerobic digestion to biogas (Klimiuk et al. 2010). For fermentation, the ideotype is likely to include greater digestibility of the lignocellulose. Other uses such as a source of biomaterials for insulation (Uihlein et al. 2008) or fiberboard (Velasquez et al. 2003) may require an optimization of processability and fiber sizes. Comparison of biomass samples harvested from the same genotypes grown at five different locations in Europe indicate that biomass composition is reasonably stable across multiple environments (Hodgson et al. 2010a) and therefore composition represents a target for optimization for different uses through plant breeding. Therefore, variation exists within Miscanthus for the optimization of bioenergy and industrial end use quality traits (Allison et al. 2011) as markets develop and mature. Other studies have also examined the potential for identifying higher value compounds in Miscanthus to increase the value within the biomass chain (Parveen et al. 2011) but the challenge is whether the market size of such compounds will be sufficient to significantly impact a large enough area of the crop and therefore number of growers.

As energy grasses are a renewable source of energy, it is important that they are sustainable and that environmental impacts are predominantly positive. Miscanthus already exhibits efficient use of resources, especially nitrogen and does not require annual fertilizer application (McCalmont et al. 2015). Indeed there is evidence that high nitrogen treatments have negative impacts on biomass yield and quality. For example, nitrogen fertilization can have a negative impact on biomass quality for thermochemical conversion with a reduction in cell wall components and an increase in ash content (Hodgson et al. 2010b). The high nitrogen efficiency of Miscanthus comes from two routes, firstly the efficient recycling of nitrogen through senescence (Mos et al. 2013) and secondly through association with nitrogen fixing bacteria (Davis et al. 2010; Keymer and Kent 2014). Water use is also already efficient compared to many crops, however because of the high biomass production improvements in water use efficiency are also considered desirable. Once established and usually after the first season Miscanthus does not require herbicide treatment even when planting into preexisting grasslands. The crop, because of the dense and efficient canopy closure, is effective at suppressing weeds which makes it ideally suited for marginal land and the bioremediation of weed infestation. There is then a potential trade‐off between productivity and suppression of long term problematic weeds and potential biodiversity of other species. Positive benefits tend to be reported during the early years of establishment, when there are gaps in the crop and also when compared to annual arable crops including those like wheat and oil seed rape which may be used for biofuels. Crop establishment was often patchy in the earlier plantings of Miscanthus, again beneficial for biodiversity, but is less common in more recent plantings as crop agronomic techniques have improved. The use of appropriate field margins may help to balance trade‐offs between productivity and biodiversity, and be another source of biomass following cutting late in the season as described above for conservation grasslands. In comparison to annual cropping systems, higher densities and diversity of soil invertebrates were observed under Miscanthus planted onto contaminated land (Hedde et al. 2013). Likewise in a large multi‐location study in Ireland differences between biodiversity in Miscanthus and conventional crops were mostly positive with higher vascular plant richness and higher solitary bee abundance and richness compared with conventional crops (Bourke et al. 2014). Perhaps, however not surprisingly, biodiversity benefits tend to be smaller and disbenefits can occur when comparisons are made to more natural or native grasslands (Dauber et al. 2015). The high biomass accumulation and leaf fall over winter help contribute to an accumulation of soil carbon. Several studies have determined soil carbon under Miscanthus to be similar to long‐term grasslands and more recent studies have been looking at the transition (Harris et al., 2014). The Miscanthus canopy can also bring about positive changes in terms of albedo and therefore contribute to global cooling measures compared to other vegetation types (Jørgensen et al. 2014). When changing land use even between different grass species, a holistic approach is needed to ensure that the benefits exceed the disbenefits. Therefore, approaches such as scenario (Harvolk et al. 2014) and opportunity mapping (Lovett et al. 2014) represent important strategies for identifying those locations where land use change is most likely to bring about predominantly positive impacts, and therefore aids policymaking.

## Concluding Remarks

Grasslands are often described as multifunctional because of the wider ecosystems services in addition to the food that they can provide, predominantly meat and milk from ruminants. The use of grassland biomass for energy and other bioproducts is an extension of this natural resource and can provide additional options for farmers and communities that are often looking for means to diversify. Moreover, large areas of poorer quality native plant grasslands are being abandoned as farmers focus their activities on improved pastures and crops, and the mechanical removal of biomass avoided by grazers for bioenergy production can be used to stimulate new plant growth with a higher nutritional value to livestock, as part of a long‐term rotation strategy with the production of energy from the removed biomass and food from the new growth. This extension in use can therefore be through the use of existing grassland vegetation or from the planting of new grassland species which can fit in with existing farming machinery and practices (as described in Table 1). Energy and other industrial uses can then provide new market opportunities for grassland farmers including to diversify income streams, to provide a means to fund land management for areas of conservation value, and be part of the solution to reduce the overall greenhouse gas emissions from livestock farming enterprises. Grasses within the built environment, like conservation biomass, represents a currently underutilized and under valorized resource, and presents an opportunity for economic and environmental impacts. Collectively grasslands and forage, energy and turf grasses form a continuum of perennial systems which can provide multiple benefits in terms of productivity and ecosystem services for those that live, work or visit them. Improving the productivity and environmental sustainability of grassland agriculture through multiple strategies, is going to be needed over the coming decades to ensure the sustainability of these important habitats and landscapes. Embracing the diversification of grass‐derived products will in many instances be an important step to ensuring grassland survival and protection from the onslaught of competition for land from urbanization and from other agricultural and forestry‐based land uses.

## Conflict of Interest

None declared.
